# p62 is Negatively Implicated in the TRAF6-BECN1 Signaling Axis for Autophagy Activation and Cancer Progression by Toll-Like Receptor 4 (TLR4)

**DOI:** 10.3390/cells9051142

**Published:** 2020-05-06

**Authors:** Mi-Jeong Kim, Yoon Min, Ji Seon Im, Juhee Son, Joo Sang Lee, Ki-Young Lee

**Affiliations:** 1 Department of Immunology, Sungkyunkwan University School of Medicine, 2066 Seobu-ro, Jangan-gu, Suwon, Gyeonggi-do 16419, Korea; kmjj0107@skku.edu (M.-J.K.); unizzzang@skku.edu (Y.M.); jsunny1319@skku.edu (J.S.I.); kimthsh@skku.edu (J.S.); 2 Department of Precision Medicine, Samsung Biomedical Research Institute, Sungkyunkwan University School of Medicine, 2066 Seobu-ro, Jangan-gu, Suwon, Gyeonggi-do 16419, Korea; lee.joosang@gmail.com; 3 Samsung Medical Center, Seoul 06351, Korea; 4 Department of Health Sciences and Technology, Samsung Advanced Institute for Health Sciences & Technology, Samsung Medical Center, Sungkyunkwan University, 81 Irwon-ro, Gangnam-gu, Seoul 06351, Korea

**Keywords:** toll-like receptor 4, p62, TRAF6, BECN1, Autophagy

## Abstract

Toll-like receptors (TLRs) induce the activation of nuclear factor kappa-light-chain-enhancer of activated B cells (NF-κB) and autophagy through the TNF (Tumor necrosis factor) receptor-associated factor 6 (TRAF6)-evolutionarily conserved signaling intermediate in Toll pathways (ECSIT) and TRAF6-BECN1 signaling axes, respectively. Having shown that p62 negatively regulates Toll-like receptor 4 (TLR4)-mediated signaling via TRAF6-ECSIT signaling axis, we herein investigated whether p62 is functionally implicated in the TRAF6-BECN1 signaling axis, thereby regulating cancer cell migration and invasion. p62 interacted with TRAF6 and BECN1, to interrupt the functional associations required for TRAF6-BECN1 complex formation, leading to inhibitions of BECN1 ubiquitination and autophagy activation. Importantly, p62-deficient cancer cells, such as p62-knockdown (p62^KD^) SK-HEP-1, p62^KD^ MDA-MB-231, and p62-knockout (p62^KO^) A549 cells, showed increased activation of autophagy induced by TLR4 stimulation, suggesting that p62 negatively regulates autophagy activation. Moreover, these p62-deficient cancer cells exhibited marked increases in cell migration and invasion in response to TLR4 stimulation. Collectively, these results suggest that p62 is negatively implicated in the TRAF6-BECN1 signaling axis, thereby inhibiting cancer cell migration and invasion regulated by autophagy activation in response to TLR4 stimulation.

## 1. Introduction

p62 (SQSTM1 gene and sequestosome-1) is a ubiquitin-binding protein and a versatile adaptor protein with multiple functions for regulating cellular events [1,2]. These include autophagic flux through the interaction with autophagic substrates, apoptosis, cellular redox regulation through the Kelch-like ECH-associated protein 1-nuclear factor (erythroid-derived 2)-like 2 (KEAP1-NRF2) pathway, adipogenesis by the interaction with extracellular-signal-regulated kinase 1 (ERK1), and nuclear factor kappa-light-chain-enhancer of activated B cells (NF-κB) signaling through the interaction with protein kinase C ζ (PKCζ) [1,2,3,4,5,6,7,8]. Although the regulatory role of p62 in inflammatory responses is controversial [9,10], it is thought to be involved in the induction of inflammatory cytokine production via TNF Tumor necrosis factor) receptor-associated factor 6 (TRAF6) polyubiquitination and, thereby, NF-κB activation [11,12]. A recent report has shown that p62 is negatively implicated in Toll-like receptor 4 (TLR4)-mediated signaling through inhibition of TRAF6-evolutionarily conserved signaling intermediate in Toll pathways (ECSIT) association and the ubiquitination of ECSIT by TRAF6 [13]. Importantly, p62^−/−^ KO mice exhibited a higher mortality rate following LPS challenge [13], suggesting that p62 might negatively regulate TLR4-mediated signaling for the activation of NF-κB.

TLRs act as the first line of host defense against microbial infections and play pivotal roles in the initiation of innate immunity and the induction of adaptive immune responses by recognizing distinct pathogen-associated molecular patterns (PAMPs) [14,15,16]. The downstream signaling cascades of TLRs are essentially mediated by several key molecules and these include myeloid differentiation primary response 88 (MyD88), TRAF6, TAK1, mitogen-activated protein kinase kinase kinase 7 (MAP3K7), and IκB kinase (IKK) complex [14,15]. Among them, TRAF6 as an E3 ubiquitin ligase and a scaffold protein plays a key role in the TLR4-mediated activation of NF-κB [17,18]. Interestingly, recent reports have demonstrated that TLR4 and TLR3 signals induce autophagy activation, and promote migration and invasion of lung cancer cells through TRAF6 ubiquitination and MAP3K7 activation [19]. Upon TLR4 stimulation, TRAF6 promotes the K63-linked ubiquitination of BECN1 to induce TLR4-mediated autophagy [19,20,21,22,23]. In addition, TLR4 signaling promotes proliferation of A549 lung cancer cells through PTGS2/COX-2 (prostaglandin-endoperoxide synthase 2 (prostaglandin G/H synthase and cyclooxygenase)) and epidermal growth factor receptor (EGFR) activation [19]. These results strongly indicate that TLR4-induced autophagy activation and cancer cell progression might be critically linked to the TRAF6-BECN1 signaling axis.

TLR4-mediated signaling induces the activation of NF-κB and autophagy induction via TRAF6-ECSIT signaling axis and TRAF6-BECN1 signaling axis, respectively [20,21,22,23,24,25,26]. Since p62 negatively regulates TLR4-mediated signals through the inhibition of TRAF6-ECSIT signaling [13], we therefore investigated whether p62 is functionally implicated in the TRAF6-BECN1 signaling axis, thereby regulating autophagy activation, and cancer cell migration and invasion induced by TLR4. We found that p62 interrupted the association of the TRAF6-BECN1 complex, and that inhibited BECN1 ubiquitination, leading to inhibition of autophagy activation induced by TLR4. Interestingly, p62-deficient cancer cells, p62-knockdown (p62^KD^) SK-HEP-1, p62^KD^ MDA-MB-231, and p62-knockout (p62^KO^) A549 cells, exhibited increases in autophagic activation, and cancer cell migration/invasion induced by TLR4 stimulation. Taken together, these results suggest that p62 negatively regulates autophagy activation, thereby functionally affecting cancer cell migration and invasion induced by TLR4.

## 2. Materials and Methods

### 2.1. Cells

Human embryonic kidney (HEK) 293T cells were obtained from the American Type Culture Collection (Ca# CRL-11268, ATCC, Manassas, VA, USA). The cells were cultured in Dulbecco’s modified Eagle’s medium (DMEM; Ca#11965092, Thermo Fisher Scientific, Waltham, MA, USA). THP-1 cells, human monocytic cells, were purchased from ATCC (Ca# TIB-202), and cultured in RPMI (Roswell Park Memorial Institute) 1640 medium (Ca#11875093, Thermo Fisher Scientific) containing 10% fetal bovine serum (FBS; Fisher Scientific HyClone, Ca#11306060), 2 mM L-glutamine (GIBCO, Ca#A2916801), 100 units/mL penicillin (GIBCO, Ca#15140122), 100 μg/mL streptomycin (GIBCO, Ca#15140122), and 5 × 10^−5^ M β-mercaptoethanol (GIBCO, Ca#21985023). Human hepatic adenocarcinoma cell line SK-HEP-1 (ATCC, HTB-52), human breast adenocarcinoma cell line MDA-MB-231 (ATCC, HTB-26), and human lung cancer cell line A549 (ATCC, CCL-185) were purchased from ATCC, and cultured in DMEM or RPMI contained with 10% FBS.

### 2.2. Generation of p62-Knockdown Cell Line

Lentivirus containing small hairpin RNA (shRNA) targeting human SQSTM1 (p62, Ca# sc-29679-V) and control shRNA lentivirus (Ca# sc-108080) were purchased from Santa Cruz Biotechnology (Santa Cruz, CA, USA). Cells were cultured in wells of a 24-well plate (2 × 10^4^ cells per well), and infected with lentivirus, according to the manufacturer’s protocol. Control (Ctrl) THP-1, Ctrl SK-HEP-1, Ctrl MDA-MB-231 cells, p62 knockdown (p62^KD^) THP-1, p62^KD^ SK-HEP-1, and p62^KD^ MDA-MB-231 cells were cultured in puromycin-containing (4–8 μg/mL) medium, and selected as previously described [21].

### 2.3. Generation of p62-Knockout Cell Line with CRISPR/Cas9

Guide RNA sequences for CRISPR/Cas9 were designed at the CRISPR design web site (http://crispr.mit.edu/), provided by the Feng Zhang Lab. Insert oligonucleotides for human SQSTM1 (p62) gRNA were 5′-CACCGTGGCTCCGGAAGGTGAAACACGG-3′/3′-CACCGAGGCCTTCCACTTTGTCAAA-5′. The p62 guide RNA targets exon 2 of p62 gene. The complementary oligonucleotides for guide RNAs (gRNAs) were annealed, and cloned into lenti CRISPR v2 vector (Addgene plasmid, Ca#52961). Lenti CRISPR v2/gRNA was transfected into A549 cells by using Lipofectamine 2000, according to the manufacturer’s instructions. After two days, cells were treated with puromycin (2 μg/mL) and cultured for three days. Colonies were isolated after two weeks, and the p62 expression in the cells was confirmed by using western blot.

### 2.4. Antibodies and Reagents

Anti-p62 antibody (Ca# ab91526) was purchased from Abcam (Cambridge, MA, USA), anti-Myc (Ca# 2276) antibody was purchased from Cell Signaling Technology (Danvers, MA, USA), and anti-Flag (Ca# F3165) and anti-HA (Ca# H3663) antibodies were purchased from Sigma-Aldrich (St Louis, MO, USA). Lipopolysaccharide (LPS, Ca#L2887), 3-methyladenine (3-MA, Ca# M9281), chloroquine (CQ, Ca# C6628), dimethyl sulfoxide (DMSO, Ca# 472301), puromycin (Ca# P9620), paraformaldehyde (Ca# P6148), Triton X-100 (Ca# T9284), gentamicin (Ca# G1272), deoxycholate (Ca# D6750), and Dulbecco’s phosphate-buffered saline (DPBS, Ca# D8537) were purchased from Sigma-Aldrich (St Louis, MO, USA). Lipofectamine 2000 (Ca# 11668-019) was purchased from Thermo Scientific (Rockford, IL, USA).

### 2.5. Plasmid Constructs

Flag-tagged TRAF6 (Ca# 21624), Flag-tagged BECN1 (Ca# 24388), and HA-tagged p62 (Ca# 28027) were purchased from Addgene (Cambridge, MA, USA). Myc-tagged ECSIT, HA-tagged Ub, and Flag-tagged ECSIT were obtained from Dr. Jae-Hyuck Shim (University of Massachusetts Medical School, USA). The constructs coding for full-length p62 with the Flag tag or Myc tag were cloned into the pCMV-3Tag-6 vector (Agilent Technologies) or pCMV-3Tag-7 vector, respectively, using HA-tagged p62 plasmid as a template. The constructs coding for full-length BECN1 with the Myc tag were cloned into the pCMV-3Tag-7 vector, using Flag-tagged BECN1 plasmid as a template. Flag-tagged TRAF6 truncated mutants and Myc-tagged BECN1 truncated mutants were generated as previously described [21,22].

### 2.6. Western Blotting Analysis and Immunoprecipitation (IP) Assays

Western blotting analysis and IP assays were carried out as previously described [13,20,21,22,23]. HEK293T cells were transfected with mock vector as control vector, Myc-tagged p62, or Flag-tagged TRAF6, and mock vector, Flag-tagged p62, and Myc-tagged BECN1 using Lipofectamine 2000. At 38 h after transfection, transfected cells were harvested, and cell lysates were immunoprecipitated with anti-Flag antibody. HEK293T cells were transfected with mock vector, Myc-tagged p62, or Flag-tagged TRAF6 wild type (WT) and Flag-tagged TRAF6 truncated mutants using Lipofectamine 2000. At 38 h after transfection, transfected cells were harvested, and cell lysates were immunoprecipitated with anti-Myc antibody. HEK293T cells were transfected with mock vector, Flag-tagged p62, or Myc-tagged BECN1 WT and Myc-tagged BECN1 truncated mutants using Lipofectamine 2000. At 38 h after transfection, transfected cells were harvested, and IP assay was performed with anti-Myc antibody. IP complexes were separated by SDS-PAGE (6–10%), and immune-probed with antibodies specific for anti-Myc or anti-Flag. HEK293T cells were transfected with mock vector, Flag-tagged TRAF6, and Myc-tagged BECN1, along with different concentrations of Myc-tagged p62, using Lipofectamine 2000. At 38 h after transfection, transfected cells were harvested, and IP assay was performed with with anti-Flag antibody. Immunoprecipitated complexes were separated by 6–10% SDS-PAGE, and probed with anti-Flag, anti-p62, or anti-BECN1 antibody. For ubiquitination assay, HEK293T cells were transfected with mock vector, Myc-tagged BECN1, Flag-tagged TRAF6, and HA-tagged Ub, along with different concentrations of Flag-tagged p62, using Lipofectamine 2000. At 38 h after transfection, transfected cells were harvested, and cell lysates were immunoprecipitated with anti-Myc antibody. Immunoprecipitated complexes were separated by 6–10% SDS-PAGE, and probed with anti-Myc, anti-HA, anti-p62, or anti-TRAF6 antibody. Control (Ctrl) THP-1 and p62^KD^ THP-1 cells, Ctrl SK-HEP-1 and p62^KD^ SK-HEP-1 cells, Ctrl MDA-MB-231 and p62^KD^ MDA-MB-231 cells, or Ctrl A549 and p62^KO^ A549 cells were treated with or without vehicle, 3MA (5mM), or CQ (10 μM), in the presence or absence of LPS (10 μg/mL), for 6 h. Whole cell lysates were immunoblotted with anti-LC3A/B antibody and anti-GAPDH as a loading control.

### 2.7. Wound-Healing and Transwell Migration Assay

A wound-healing assay was carried out as previously described [21,22]. Ctrl SK-HEP-1 and p62^KD^ SK-HEP-1 cells, Ctrl MDA-MB-231 and p62^KD^ MDA-MB-231 cells, or Ctrl A549 and p62^KO^ A549 cells were cultured in 12-well plates, and inoculated to confluence. Cell monolayers were gently scratched by using a sterile yellow Gilson-pipette tip to make a wide gap (approximately 400 μm). Cells were washed with culture medium, and floating cells and debris were removed from plates. Cells were treated with vehicle (DMSO, <0.2% in DMEM culture medium), 3-MA (5 mM), or CQ (10 μM) in the presence or absence of LPS (10 μg/mL), and images were captured after different times as indicated in each experiment. Transwell inserts (8μm pore; Corning, 3422) were sited into wells for cell migration assay. 5 × 10^4^ cells per well were suspended in culture medium (DMEM) including vehicle, 3-MA (5 mM), or CQ (10 μM) in the presence or absence of LPS (10 μg/mL), and placed into the top chambers of the 24-transwell plates. Culture medium, DMEM contained 10% FBS, was added to the bottom chambers. After an overnight incubation, the non-migrated cells to be remained in the top chamber were removed. The migrated cells to be existed in the bottom chamber were fixed. To visualize the nuclei, cells were stained by using crystal violet. All experiments were performed in triplicate. The experiments were repeated twice times.

### 2.8. Reverse Transcription-Quantitative Polymerase Chain Reaction (RT-qPCR)

Control (Ctrl) and p62^KO^ A549 cells were treated with or without 10 μg/mL LPS for 6 h. Total RNA was extracted from cells using an RNA isolation kit (A&A Biotechnology, Gdynia, Poland) according to the manufacturer’s protocol. cDNA was obtained by RT using a amfiRivert II cDNA Synthesis Master Mix (genDEPOT, R550), according to the manufacturer’s protocol. Primers for hIL-6 (PPH 00560C), hMMP2 (PPH 00151B), and hCCL2 (PPH 00192F) were purchased from Qiagen, Inc. (Chatsworth, CA, USA). Fluorescence detection was performed using the ABI PRISM 7700 Sequence Detector (PerkinElmer; Applied Biosystems; Thermo Fisher Scientific, Inc.). The mRNA expressions were calculated and normalized to the level of GAPDH.

### 2.9. Statistical Analysis

In vitro data are expressed as mean ±SEM of triplicate samples. Statistical significance of experiments was analyzed by using ANOVA or Student’s *t*-test using GraphPad Prism 5.0 (GraphPad Software, San Diego, CA, USA).

## 3. Results

### 3.1. P62 Interacts with TRAF6 and BECN1

**Figure 1:**
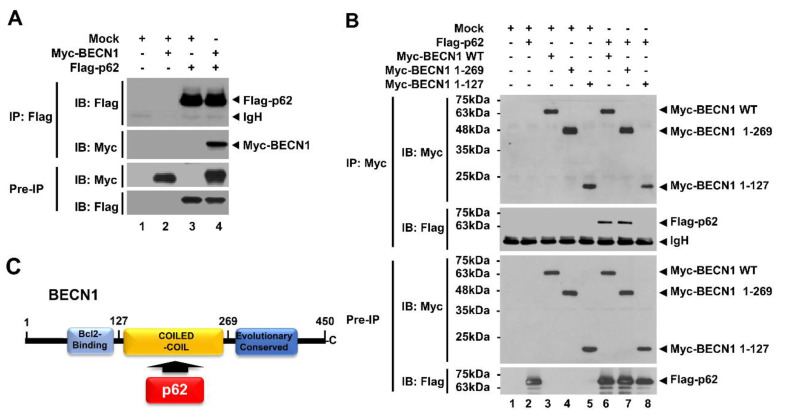
p62 interacts with BECN1 proteins. (**A**) HEK293T cells were transfected with mock control vector, Flag-tagged p62, or Myc-tagged BECN1, as indicated. Transfected cells were harvested, and cell lysates were immunoprecipitated with anti-Flag antibody and probed with anti-Myc or anti-Flag antibody. (**B**) HEK293T cells were transfected with mock vector, Flag-tagged p62, or Myc-tagged BECN1 WT and Myc-tagged BECN1 truncated mutants. Transfected cells were harvested, and cell lysates were immunoprecipitated with anti-Myc antibody. Immunoprecipitated complexes were separated by SDS-PAGE, and probed with anti-Myc or anti-Flag antibody. (**C**) A schematic view of the molecular interaction between BECN1 and p62.

It has been reported that p62 plays an inhibitory role in TLR4 signaling through interrupting the association of TRAF6 with ECSIT, eventually leading to inhibition of NF-κB activation [13]. TRAF6 is an essential regulator for the induction of NF-κB and autophagy in TLR4 signaling [14,15,16,17,18]. Based on these earlier reports, we asked whether p62 is involved in TLR4-induced activation of autophagy. To investigate this, we first examined the molecular association between p62 and autophagy regulator proteins, such as TRAF6 and BECN1, in TLR4 signaling. Myc-tagged p62 or Flag-tagged p62 expressing vector were transfected into HEK293T cells along with Flag-tagged TRAF6 or Myc-tagged BECN1 expressing vector, and then immunoprecipitation (IP) was performed with anti-Flag antibody. Consistent with a previous report [13], Flag-tagged TRAF6 precipitated with Myc-tagged p62 (Supplementary Materials Figure S1A, lane 4). Additionally, Flag-tagged p62 precipitated with Myc-tagged BECN1 (Figure 1A, lane 4). To determine the specific binding site of p62 to TRAF6 or BECN1, truncated mutants of TRAF6 or BECN1 were generated, and IP assay was performed between p62 and these truncated proteins (Supplementary Materials Figure S2A,B). As shown in Supplementary Materials Figure S3, Flag-tagged TRAF6 wild type (WT) and Flag-tagged TRAF6 truncated mutants were significantly precipitated with Myc-tagged p62 (Supplementary Materials Figure S3A, lane 6-8), suggesting that p62 interacts with the TRAF-C domain of TRAF6 (Supplementary Materials Figure S3B). The results were consistent with a previous report [13]. As well, Myc-tagged BECN1 WT and Myc-tagged BECN1 1-269 truncated mutant were precipitated with Flag-tagged p62 (Figure 1B, lane 6 and 7), whereas no significant interaction could be observed with Myc-tagged BECN1 1-127 truncations (Figure 1B, lane 8), indicating that p62 interacts with the coiled-coil domain of BECN1 (Figure 1C).

### 3.2. p62 Interrupts the Association of BECN1-TRAF6 and Inhibits the Ubiquitination of BECN1 Induced by TRAF6

**Figure 2:**
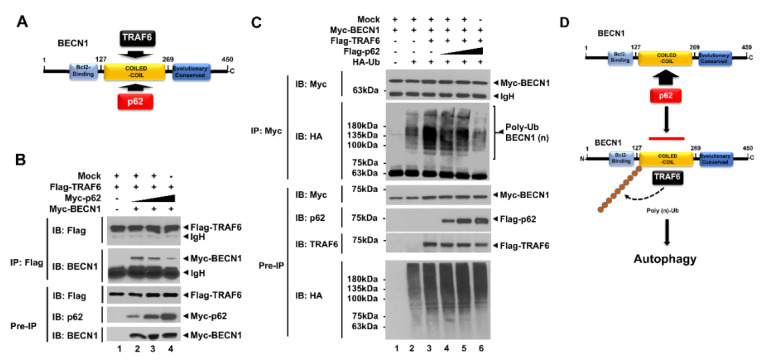
p62 interrupts the association of TNF (Tumor necrosis factor) receptor-associated factor 6 (TRAF6)-BECN1 complex and inhibits the ubiquitination of BECN1. (**A**) TRAF6 and p62 interact with the coiled-coil domain of BECN1. (**B**) HEK293T cells were transfected with mock vector, Flag-tagged TRAF6, and Myc-tagged BECN1, along with different concentrations of Myc-tagged p62, as indicated. Transfected cells were harvested, and cell lysates were immunoprecipitated with anti-Flag antibody and probed with anti-Flag, anti-p62, or anti-BECN1 antibody. (**C**) HEK293T cells were transfected with mock vector, Myc-tagged BECN1, Flag-tagged TRAF6, and HA-tagged Ub, along with different concentrations of Flag-tagged p62, as indicated. Transfected cells were harvested, and cell lysates were immunoprecipitated with anti-Myc antibody and probed with anti-Myc, anti-HA, anti-p62, or anti-TRAF6 antibody. (**D**) A schematic model for how p62 interrupts the association of TRAF6-BECN1 complex and inhibits the ubiquitination of BECN1. TRAF6 interacts with the coiled-coil domain of BECN1 and induces the ubiquitination of BECN1, leading to autophagy activation. Simultaneously, p62 can interact with the coiled-coil domain of BECN1, and that inhibits the interaction of TRAF6 to BECN1 and the ubiquitination of BECN1.

A previous study showed that TRAF6 interacts with the BECN1 and induces the ubiquitination of BECN1, leading to induction of autophagy [21]. Consistently, Flag-tagged TRAF6 was significantly precipitated with Myc-tagged BECN1 (Supplementary Materials Figure S4A). Importantly, TRAF6 interacted with the coiled-coil domain of BECN1 [21], as depicted in Supplementary Materials Figure S4B. As shown in Figure 1B,C, p62 interacted with the coiled-coil domain of BECN1, which was the same binding domain as TRAF6 [21]. Therefore, we raised the possibility that p62 affects the molecular association of TRAF6-BECN1, thereby inhibiting BECN1 ubiquitination, as depicted in Figure 2A. To examine this possibility, we performed a competitive binding assay with different concentrations of p62. Flag-tagged TRAF6 and Myc-tagged BECN1 vectors were transfected into HEK293T cells along with different concentrations of Myc-tagged p62 vectors, as indicated Figure 2B. IP was then performed with anti-Flag antibodies in cell lysates. Correlating to increases of Myc-tagged p62 vector, the interactions between Flag-tagged TRAF6 and Myc-tagged BECN1 were significantly attenuated (Figure 2B, lane2-4; IB: BECN1 in IP with Flag-tagged TRAF6), suggesting that p62 interrupts the molecular association between TRAF6 and BECN1.

Since TRAF6 interacted with BECN1 and induced its ubiquitination [19,20,21,22], we examined whether inhibiting the interaction between TRAF6 and BECN1 by p62 affects BECN1 ubiquitination. To do that, Myc-tagged BECN1, Flag-tagged TRAF6, and HA-tagged Ub vectors were transfected into HEK293T cells along with different concentrations of Flag-tagged p62, and then IP was performed with anti-Myc antibodies. The marginal ubiquitination of BECN1 could be seen in the absence of Flag-tagged TRAF6 (Figure 2C, lane 2), whereas a marked increase could be seen in the presence of Flag-tagged TRAF6 (Figure 2C, lane 3). Based on increasing Flag-tagged p62 expression, the ubiquitination of BECN1 was gradually attenuated (Figure 2C, lane 4-6). These results suggest that p62 interrupts the molecular interaction between TRAF6 and BECN1, and that in turn inhibits the ubiquitination of BECN1 induced by TRAF6, as depicted in Figure 2D.

Studies have shown that the TRAF6-induced ubiquitination of BECN1 plays a key role in TLR-induced autophagy activation, thereby functionally implicating cancer progression and migration [19,21]. We found that p62 interrupted the association between BECN1 and TRAF6, and inhibited the ubiquitination of BECN1 (Figure 2B,C). Therefore, we explored the functional role of p62 in autophagy induction, cancer progression, and migration, all of which are regulated by TLR4 signaling [19,21], as depicted in Figure 2D.

### 3.3. p62-Knockdown THP-1 (p62^KD^ THP-1), SK-HEP-1 (p62^KD^ SK-HEP-1), and MDA-MB 231 (p62^KD^ MDA-MB-231) Cells Exhibit Elevated Autophagy Activation Induced by TLR4 Stimulation

**Figure 3:**
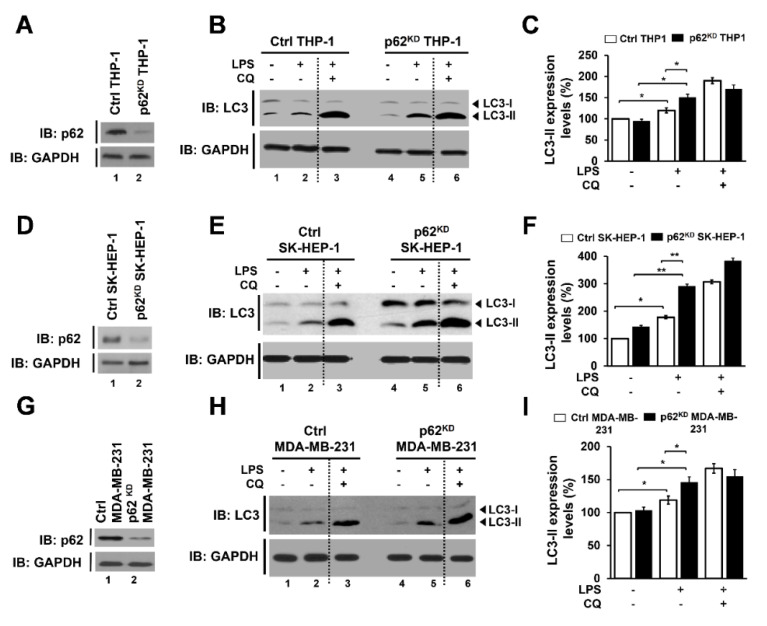
p62-deficient cells, p62^KD^ THP-1, p62^KD^ SK-HEP-1, and p62^KD^ MDA-MB-231 cells, exhibit enhanced autophagy activation in response to TLR4 stimulation. (**A**) p62^KD^ THP-1 cells were generated, and the knockdown efficacy of p62 was confirmed with anti-p62 antibody. (**B**,**C**) Ctrl and p62^KD^ THP-1 cells were treated with or without vehicle or CQ (10 μM), in the presence or absence of LPS (10 μg/mL), for 6 h. Whole cell lysates were immunoblotted with anti-LC3A/B antibody and anti-GAPDH antibody as a loading control (**B**). The LC3II levels were analyzed with Image J program (**C**). Data shown are averages from a minimum of 3 independent experiments (± SEM). *, *p* < 0.05. (**D**) p62^KD^ SK-HEP-1 cells were generated, and the knockdown efficacy of p62 was confirmed with anti-p62 antibody. (**E**,**F**) Ctrl and p62^KD^ SK-HEP-1 were treated with or without vehicle or CQ, in the presence or absence of LPS. Whole cell lysates were immunoblotted with anti-LC3A/B and anti-GAPDH antibodies (**E**). The LC3II levels were analyzed with Image J program (**F**). Data shown are averages from a minimum of 3 independent experiments (± SEM). *, *p* < 0.05 and **, *p* < 0.01. (**G**) p62^KD^ MDA-MB-231 cells were generated, and the knockdown efficacy of p62 was confirmed with anti-p62 antibody. (**H**,**I**) Ctrl and p62^KD^ MDA-MB-231 were treated with or without vehicle or CQ, in the presence or absence of LPS. Whole cell lysates were immunoblotted with anti-LC3A/B and anti-GAPDH antibodies (**H**). The LC3II levels were analyzed with Image J program (**I**). Data shown are averages from a minimum of 3 independent experiments (± SEM). * *p* < 0.05.

To investigate the functional role of p62 in autophagy activation induced by TLR4 stimulation, we generated p62-knockdown THP-1 (p62^KD^ THP-1 cells) by using the lentivirus containing shRNA targeted to p62, as described in Materials and Methods. The efficiency of p62 knockdown was significant compared to control cells (Figure 3A, lane 1 versus lane 2). Control (Ctrl) THP-1 and p62^KD^ THP-1 cells were treated with or without LPS and an autophagy inhibitor, chloroquine (CQ), as indicated in Figure 3B, and autophagy activation was assessed by western blotting with anti-LC3 antibody. Upon LPS stimulation, the level of LC3-II increased in both cell lines, (Figure 3B, lane 1 versus lane 2 in Ctrl THP-1 and lane 4 versus lane 5 in p62^KD^ THP-1), but the increase in LC3-II was significantly higher in p62^KD^ THP-1 cells than in Ctrl THP-1 cells (Figure 3B, lane 2 versus lane 5; Figure 3C, open bars versus closed bars in LPS). As expected, CQ treatment induced the marked accumulation of LC3-II in both cell lines (Figure 3B, lane 3 and lane 6; Figure 3C, open bars versus closed bars in LPS plus CQ), suggesting that p62 negatively regulates autophagy activation induced by TLR4 stimulation. 

To investigate further the role of p62 in activating autophagy, we generated p62-knockdowns in two cancer cell lines, p62^KD^ SK-HEP-1 and p62^KD^ MDA-MB 231 cells, as described in Materials and Methods. The efficacy of p62 knockdown in SK-HEP-1 and MDA-MB-231 cells was significant as compared to control cells (Figure 3D in SK-HEP-1 and 3G in MDA-MB-231 cells, lane 1 versus lane 2). In similar fashion to p62^KD^ THP-1 cells (Figure 3B,C), the levels of LC3-II were significantly enhanced in p62^KD^ SK-HEP-1 and p62^KD^ MDA-MB-231 cells in the presence of LPS, as compared to their controls (Figure 3E,F in SK-HEP-1 lane 2 versus lane 5 and open bars versus closed bars in LPS treated; Figure 3H,I in MDA-MB-231, lane 2 versus lane 5 and open bars versus closed bars in LPS treated). These results suggest that p62 negatively regulates autophagy activation induced by TLR4 stimulation, presumably by the inhibition of the ubiquitination of BECN1 as demonstrated in Figure 2D.

### 3.4. p62-Deficient Cancer Cells Exhibit Increased Cancer Cell Migration and Invasion, Induced by TLR4 Stimulation

**Figure 4:**
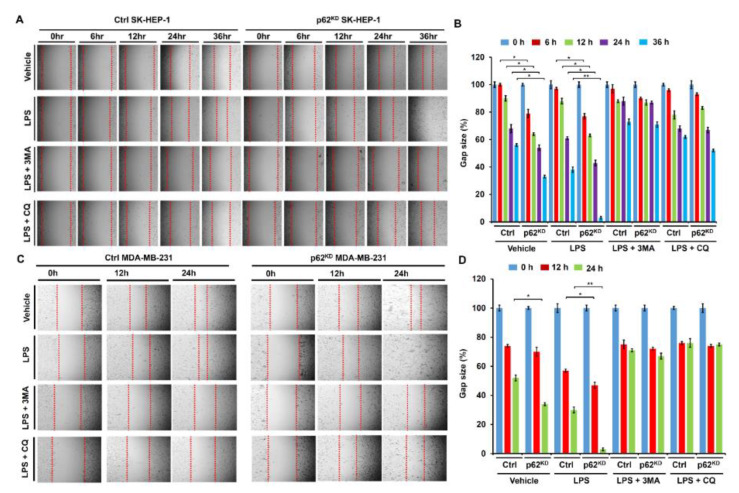
p62^KD^ SK-HEP-1 and p62^KD^ MDA-MB-231 cells exhibit increased cell migration in response to TLR4 stimulation. (**A**,**B**) Ctrl and p62^KD^ SK-HEP-1 cells were seeded into 12-well cell culture plates. Confluent monolayers were scraped with a sterile yellow Gilson-pipette tip, and the wound was then treated with vehicle (DMSO, <0.2% in culture medium), LPS (10 μg/mL), 3-MA (5 mM) plus LPS (10 μg/mL), and CQ (10 μM) plus LPS (10 μg/mL) for different time periods, as indicated. A representative experiment is shown (**A**). The residual gap between the migrating cells from the opposing wound edge was expressed as a percentage of the initial scraped area (± SEM, *n* = 3) (**B**). *, *p* < 0.05 and **, *p* < 0.01. (**C**,**D**) Ctrl and p62^KD^ MDA-MB-231 were seeded into 12-well cell culture plates. Confluent monolayers were scraped with a sterile yellow Gilson-pipette tip, and the wound was then treated with vehicle (DMSO, <0.2% in culture medium), LPS (10 μg/mL), 3-MA (5 mM) plus LPS (10 μg/mL), and CQ (10 μM) plus LPS (10 μg/mL) for different time periods, as indicated. A representative experiment is shown (**C**). The residual gap between the migrating cells from the opposing wound edge was expressed as a percentage of the initial scraped area (± SEM, *n* = 3) (**D**). * *p* < 0.05 and ** *p* < 0.01.

**Figure 5:**
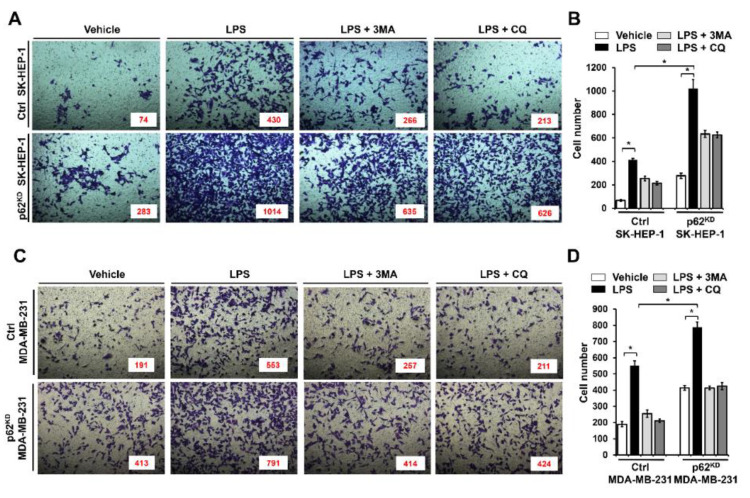
p62^KD^ SK-HEP-1 and p62^KD^ MDA-MB-231 cells exhibit increased invasiveness in response to TLR4 stimulation. (**A**,**B**) Ctrl and p62^KD^ SK-HEP-1 cells were suspended in DMEM culture medium including vehicle, LPS (10 μg/mL), 3-MA (5 mM) plus LPS (10 μg/mL), and CQ (10 μM) plus LPS (10 μg/mL). Cells were placed into the top chambers of 24-transwell plates and incubated for overnight. Fixed cells were stained by using crystal violet (**A**). Numbers of migrated cells were counted, and results are represented as mean ± SEM (**B**). * *p* < 0.05. (**C**,**D**) Ctrl and p62^KD^ MDA-MB-231 cells were suspended in culture medium of RPMI including vehicle, LPS (10 μg/mL), 3-MA (5 mM) plus LPS (10 μg/mL), and CQ (10 μM) plus LPS (10 μg/mL). Cells were placed into top chambers of 24-transwell plates and further incubated for overnight. Fixed cells were stained with crystal violet (**C**). Numbers of migrated cells were counted, and results are represented as mean ± SEM (**D**). * *p* < 0.05.

Having shown that p62 negatively regulated autophagy activation, we asked whether the inhibitory effect was functionally associated with cancer cell migration and invasion. To do that, migration and invasion assays were performed in p62^KD^ SK-HEP-1 and p62^KD^ MDA-MB-231 cells. Ctrl SK-HEP-1 and p62^KD^ SK-HEP-1 were treated with vehicle, LPS, LPS plus a 3-methyladenine (3-MA) autophagy inhibitor, and LPS plus a CQ autophagy inhibitor, and then wound healing assay was performed. Based on LPS treatment, cancer cell migratory behavior was significantly higher in p62^KD^ SK-HEP-1 than the Ctrl SK-HEP-1 cells in a time dependent manner (Figure 4A,B, Ctrl versus p62^KD^ SK-HEP-1 in LPS treatment). These results were consistently observed in Ctrl and p62^KD^ MDA-MB-231 cells (Figure 4C,D, Ctrl versus p62^KD^ MDA-MB 231 in LPS treatment). As expected, marked attenuations could be seen in co-treatments with 3-MA or CQ (Figure 4A,B, Ctrl versus p62^KD^ SK-HEP-1 in LPS plus 3-MA or CQ: Figure 4C,D, Ctrl versus p62^KD^ MDA-MB-231 in LPS plus 3-MA or CQ). We next assessed invasiveness following TLR4 stimulation. Ctrl SK-HEP-1 and p62^KD^ SK-HEP-1 were treated with vehicle, LPS, LPS plus 3-MA, and LPS plus CQ, and then transwell migration assay was performed. Similar to the wound healing assay, progressive invasiveness was significantly higher in p62^KD^ SK-HEP-1 than in Ctrl SK-HEP-1 in the presence of LPS (Figure 5A,B, Ctrl versus p62^KD^ SK-HEP-1 in LPS treatment). Consistent results were observed in Ctrl and p62^KD^ MDA-MB-231 cells (Figure 5C,D, Ctrl versus p62^KD^ MDA-MB-231 in LPS treatment).

**Figure 6:**
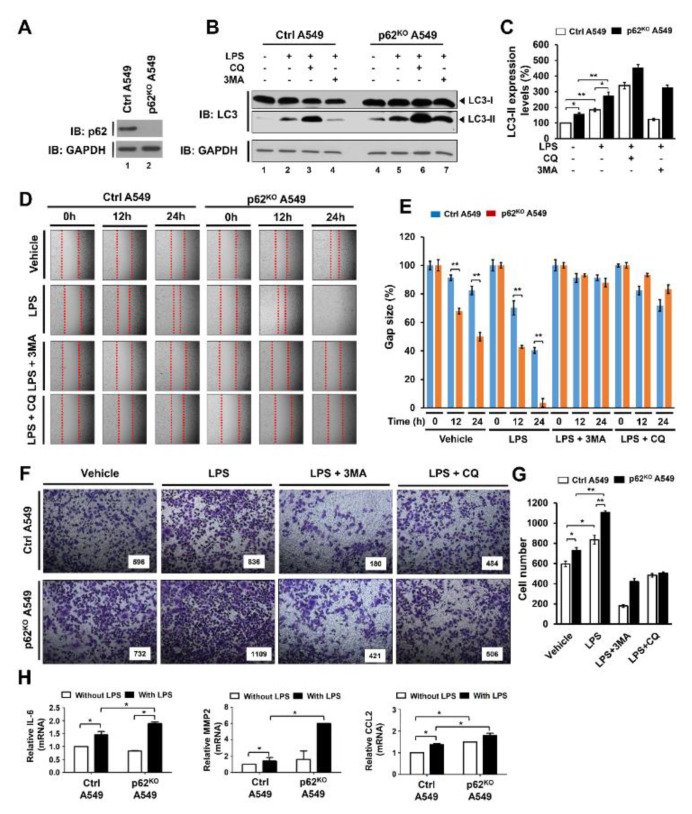
p62^KO^ A549 cells exhibited increased autophagy activation, migration, and invasion in response to TLR4 stimulation. (**A**) p62^KO^ A549 cells were generated, and the knockout efficacy of p62 was confirmed with anti-p62 antibody. (**B**,**C**) Ctrl and p62^KO^ A549 cells were treated with or without vehicle, CQ (10 μM), or 3-MA (5 mM), or, in the presence or absence of LPS (10 μg/mL), for 6 h. Whole cell lysates were immunoblotted with anti-LC3A/B antibody and anti-GAPDH antibody as a loading control (**B**). The LC3II levels were analyzed with Image J program (right, histogram) (**C**). Data shown are averages from a minimum of 3 independent experiments (± SEM). * *p* < 0.05 and ** *p* < 0.01. (**D**,**E**) Ctrl and p62^KO^ A549 cells were seeded into 12-well cell culture plates. Confluent monolayers were scraped with a sterile yellow Gilson-pipette tip to make wounds, and then incubated with vehicle (DMSO, <0.2% in culture medium), LPS (10 μg/mL), 3-MA (5 mM) plus LPS (10 μg/mL), and CQ (10 μM) plus LPS (10 μg/mL) for different time periods. A representative experiment is represented (**D**). The residual gap between the migrating cells from the opposing wound edge was represented as a percentage of the initial scraped area (± SEM, *n* = 3) (**E**). ** *p* < 0.01. (**F**,**G**) Ctrl and p62^KO^ A549 cells were suspended in RPMI medium including vehicle, LPS (10 μg/mL), 3-MA (5 mM) plus LPS (10 μg/mL), and CQ (10 μM) plus LPS (10 μg/mL), and placed to the top chambers of 24-transwell plates. After an overnight incubation, cells were fixed and stained with crystal violet (**F**). Number of migrating cell were counted, and results are presented as mean ± SEM of 3 independent experiments (**G**). * *p* < 0.05 and ** *p* < 0.01. (**H**) Control (Ctrl) and p62^KO^ A549 cells were treated with or without 10 μg/mL LPS for 6 h. Total RNA was extracted, cDNA was obtained, as described in materials and methods, and RT-qPCR analysis performed with specific primers, such as hIL-6, hMMP2, and hCCL2. * *p* < 0.05.

To verify the above results, we generated p62-knockout (p62^KO^) A549 cells using Crispr cas9 (Figure 6A). Upon TLR4 stimulation, the levels of LC3-II were significantly increased in both Ctrl A459 and p62^KO^ A549 cells (Figure 6B, lane 1 versus lane 2 in Ctrl A549 and lane 4 versus lane 5 in p62^KO^ A549 cells). Moreover, the LC3-II ratio relative to control was significantly higher in p62^KO^ A549 cells than in Ctrl A549 (Figure 6C, closed bar versus open bar in LPS). As expected, treatments of autophagy inhibitor CQ or 3-MA induced increased or decreased LC3-II levels, respectively (Figure 6B, lane 3 and lane 4 in Ctrl; lane 6 and 7 in p62^KO^ A549). Consistent with results of p62^KD^ SK-HEP-1 and p62^KD^ MDA-MB 231, the cell migration and invasion activities induced by LPS were markedly enhanced in p62^KO^ A549 cells compared to controls, whereas significant attenuations were observed in co-treatments with 3-MA or CQ (Figure 6D,E, Ctrl versus p62^KO^ A549 in LPS treatment; Figure 6F,G, Ctrl versus p62^KO^ A549 in LPS treatment). It has been previously reported that TLR4-induced autophagy activation promoted migration and invasion of lung cancer by induction of chemokines and immunosuppressive factors including CCL2, CCL20, IL-6, VEGFA, and MMP2 [19,27,28]. Therefore, we evaluated the production of IL-6, MMP2, and CCL2 in Ctrl and p62^KO^ A549 cells in the presence or absence of LPS stimulation. Consistent with the earlier report, the levels of IL-6 mRNA, MMP2 mRNA, CCL2 mRNA were significantly elevated in Ctrl A549 cells treated with LPS (Figure 6H, without LPS versus with LPS in Ctrl A549). Interestingly, these levels were markedly higher in p62^KO^ A549 cells than in Ctrl A549 cells under LPS stimulation (Figure 6H, Ctrl versus p62^KO^ A549 with LPS). Collectively these results suggest that p62 negatively regulates autophagy activation, cancer cell migration and invasion induced by TLR4 stimulation.

## 4. Discussion

**Figure 7:**
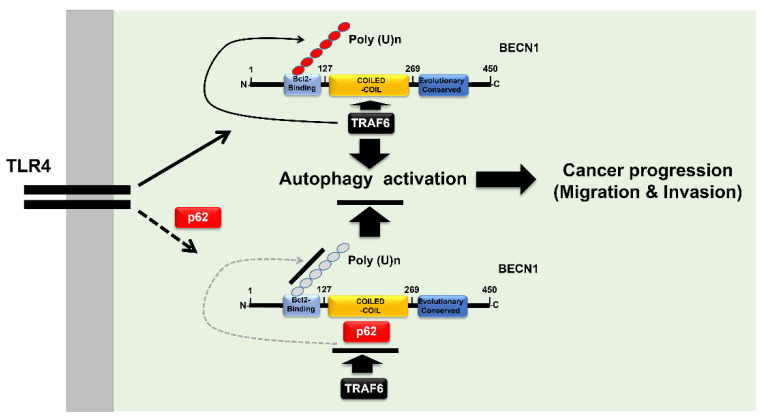
p62 is negatively involved in autophagy activation, and cancer cell migration and invasion in response to TLR4 stimulation. Engagement of TLR4 ligand leads to the association of TRAF6-BECN1 complex, and that induces the ubiquitination of BECN1. BECN1 ubiquitination induces the activation of autophagy, thereby potentially regulating cancer progression, via migration and invasion, as depicted in the upper panel. However, the interaction between p62 and BECN1 inhibits the association of TRAF6 to BECN1, and that inhibits the ubiquitination of BECN1, leading to inhibitions of autophagy activation, and cancer cell migration and invasion, as depicted in the lower panel.

In this study, we demonstrate that p62, which is a known negative regulator for the activation of autophagy by TLR4 signaling, inhibits the ubiquitination of BECN1 mediated by TRAF6. We demonstrate this occurs through an interruption to the molecular association between TRAF6 and BECN1 and, thereby, involves cancer cell migration and invasion, activities that are facilitated by autophagy, as depicted in Figure 7. Through the biochemical studies, we found that p62 competitively interacted with the coiled-coil domain of BECN1 affecting binding to TRAF6, and that induced an attenuation of BECN1 ubiquitination. Levels of LC3-II revealed increased autophagy induction in the presence of TLR4 stimulation by p62^KD^ THP-1 and p62^KD^ cancer cells. More importantly, these p62-deficient cancer cells, including p62^KO^ A549 cells, showed enhanced migration and invasion in response to TLR4 stimulation. Taken together, these results suggest that p62 is negatively implicated in the activation of autophagy by TLR4 signaling, thereby demonstrating an involvement in cancer cell invasiveness facilitated by autophagy induction.

Accumulating evidence suggests that TLR signaling might be critical for induction and activation of autophagy, thereby facilitating cancer cell migration and invasion [19,21]. Among signal cascades, TRAF6 plays a pivotal role in NF-κB activation and autophagy induction through the activation of TLR downstream molecules and the ubiquitination of BECN1, respectively [19,20,21,22,23,24,25,26]. A previous study demonstrated that p62 attenuated the ubiquitination of ECSIT, which is known as one of the regulators in TLR4-mediated signaling for NF-κB activation, induced by TRAF6 [13]. Interestingly, we found in the current study that p62 interacted with BECN1, as well as TRAF6. The molecular associations between p62 and BECN1 or TRAF6 revealed that p62 interacted with the coiled-coil domain of BECN1, which also surprisingly served as the interaction site for TRAF6 [21], possibly explaining the inhibitory mechanism of p62 in the association of TRAF6-BECN1. p62 interference in TRAF6-BECN1 complex formation, eventually induced the attenuation of BECN1 ubiquitination by TRAF6.

Another important finding in the current study was that p62 regulated cancer cell invasiveness was facilitated by TLR4 stimulation through autophagy activation. Although there is some controversy over whether autophagy activation is either positively or negatively involved in cancer metastasis [29,30,31,32,33], recent studies have demonstrated that TLRs induced cancer cell migration and invasion through facilitating autophagy induction, which was mechanistically associated with the TRAF6-BECN1 signaling axis [19,21]. Consistent with these reports, we found that p62-deficient cancer cells, exhibited elevated autophagy activation, cancer cell migration, and invasiveness in response to TLR4 stimulation. Moreover, TCGA (The Cancer Genome Atlas) data revealed that the expression of p62 was significantly lower in primary tumors, such as colorectal cancer, breast cancer, prostate adenocarcinoma/prostate cancer, and bladder cancer/bladder urothelial carcinoma, than those of normal primary cells (Supplementary Materials Figure S5A–D). In addition, metastatic tumors, such as clear cell renal cell carcinoma, kidney cancer, and colorectal cancer, showed greater downregulation of p62, compared to primary tumors (Supplementary Materials Figure S6A,B). These results strongly suggest that p62 may be negatively associated with autophagy activation in cancer cells induced by TLR4 stimulation, and thereby be a key regulator of cancer cell metastasis. 

In summary, we propose a molecular mechanism for, and functional effects of, p62 in autophagy activation and cancer progressions induced by TLR4 stimulation, as depicted in Figure 7. There are some controversies related to p62 and its involvement in inflammatory versus anti-inflammatory responses [9,10,11,12]. Considering p62 is a versatile adaptor protein with multiple cellular functions [1,2,3,4,5,6,7,8], the outstanding issues need to be clarified in the near future. Our results contribute to a growing understanding of the multi-functional role of p62 in autophagy and cancer progression. This new understanding may prove useful for the development of promising therapeutic approaches for treatment of inflammatory diseases and cancers in certain pathological conditions.

## Supplementary Materials

The following are available online at https://www.mdpi.com/2073-4409/9/5/1142/s1, Figure S1: TRAF6 interacts with p62.; Figure S2: Generations of TRAF6 or BECN1 truncated mutants.; Figure S3: p62 interacts with the TRAF-C domain of TRAF6.; Figure S4: TRAF6 interacts with the coiled-coil domain of BECN1.; Figure S5: The association between SQSTM1/p62 expression and primary tumors.; Figure S6: The association between SQSTM1/p62 expression and tumor metastasis.
