# Supplementary material for: p62 is Negatively Implicated in the TRAF6-BECN1 Signaling Axis for Autophagy Activation and Cancer Progression by Toll-Like Receptor 4 (TLR4)
Source: Cells. 2020 May 6;9(5):1142. doi: 10.3390/cells9051142 (PMC7290749; doi:10.3390/cells9051142)
Supplement: Supplementary file 1 [file cells-09-01142-s001.zip › Supplementary information (Kim et al., 2020 Cells).docx]

**Supplementary information**

**1. Supplementary materials and methods**

*1.1. Human Cancer Metastasis Dataset analysis*

The association between SQSTM1/p62 expression and different cancers or cancer metastasis was analyzed using HCMDB Human Cancer Metastasis Dataset (<https://hcmdb.i-sanger.com/>).

*1.2. Western blotting analysis*

HEK293T cells were transfected with Mock as a control vector, Myc-tagged BECN1, or Flag-tagged TRAF6 using Lipofectamine 2000. At 38 h after transfection, transfected cells were harvested, and cell lysates were immunoprecipitated with anti-Flag antibody. Immunoprecipitated complexes were separated by (6–10) % SDS-PAGE, and probed with anti-Myc or anti-Flag antibody.

*1.3. Reverse transcription-quantitative polymerase chain reaction (RT-qPCR)*

RT-qPCR was performed as previously described [1]. Briefly, control (Ctrl) and p62^KO^ A549 cells were treated with or without 1mg/ml LPS for 6 hr. Total RNA was extracted from cells using an RNA isolation kit (A&A Biotechnology, Gdynia, Poland) according to the manufacturer's protocol. cDNA was obtained by RT using a amfiRivert II cDNA Synthesis Master Mix (genDEPOT, R550), according to the manufacturer's protocol. Primers for hIL-6 (PPH 00560C), hMMP2 (PPH 00151B), and hCCL2 (PPH 00192F) were purchased from Qiagen, Inc. Fluorescence detection was performed using the ABI PRISM 7700 Sequence Detector (PerkinElmer; Applied Biosystems; Thermo Fisher Scientific, Inc.). The mRNA expressions were calculated [2] and normalized to the level of GAPDH.

**References**

1. Wang T, Zhan Q, Peng X, Qiu Z, Zhao T. CCL2 influences the sensitivity of lung cancer A549 cells to docetaxel. Oncol Lett. 2018 Jul;16(1):1267-1274.
2. Livak KJ, Schmittgen TD. Analysis of relative gene expression data using real-time quantitative PCR and the 2(-Delta Delta C(T)) Method. Methods. 2001 Dec;25(4):402-8.

**Figure S1.** Generations of TRAF6 or BECN1 truncated mutants. **(A)**. Flag-tagged TRAF6 truncated mutants, Flag-tagged TRAF6 260-522 and Flag-tagged TRAF6 349-522, were generated using Flag-tagged TRAF6 wild type (WT) vector as a template. **(B)** Myc-tagged BECN1 truncated mutants, Myc-tagged BECN1 1-269 and Mcy-tagged BECN1 1-127, were generated using Myc-tagged BECN1 WT as a template.

**Figure S2**. TRAF6 interacts with BECN1. HEK293T cells were transfected with Mock as a control vector, Myc-tagged BECN1, or Flag-tagged TRAF6 using Lipofectamine 2000. At 38 h after transfection, transfected cells were harvested, and cell lysates were immunoprecipitated with anti-Flag antibody. Immunoprecipitated complexes were separated by (6–10) % SDS-PAGE, and probed with anti-Myc or anti-Flag antibody.

**Figure S3.** RT-qPCR analysis of hIL-6, hMMP2, and hCCL2 in p62^KO^ A549 cells induced by TLR4 stimulation. (**A-C**). Control (Ctrl) and p62^KO^ A549 cells were treated with or without 1mg/ml LPS for 6 hr. Total RNA was extracted, cDNA was obtained, as described in supplementary materials and methods, and RT-qPCR analysis performed with specific primers, such as hIL-6 (**A**), hMMP2 (**B**), and hCCL2 (**C**). **P*<0.05.

**Figure S4.** The association between SQSTM1/p62 expression and primary tumors. (**A-D**) The expression of SQSTM1/p62 was compared between primary normal and primary tumors, such as colorectal cancer (**A**), breast cancer (**B**), prostate adenocarcinoma and prostate cancer (**C**), and bladder cancer and bladder urothelial carcinoma (**D**), by using HCMDB Human Cancer Metastasis Dataset (<https://hcmdb.i-sanger.com/>).

**Figure S5.** The association between SQSTM1/p62 expression and tumor metastasis. (**A and B**) The expression of SQSTM1/p62 was compared between primary tumor and metastasis tumors, such as clear cell renal cell carcinoma and kindey cancer (**A**), and colorectal cancer (**B**), by using HCMDB Human Cancer Metastasis Dataset (<https://hcmdb.i-sanger.com/>).
