# Supplementary figures and images for: p62 is Negatively Implicated in the TRAF6-BECN1 Signaling Axis for Autophagy Activation and Cancer Progression by Toll-Like Receptor 4 (TLR4)
Source: Cells. 2020 May 6;9(5):1142. doi: 10.3390/cells9051142 (PMC7290749; doi:10.3390/cells9051142)

**A**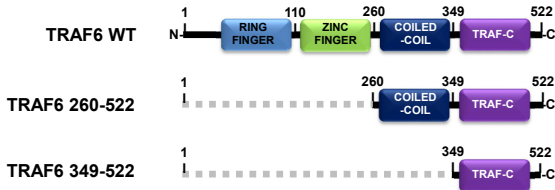**B**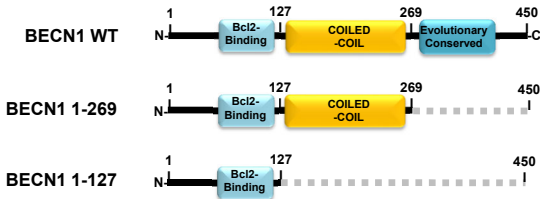

***Supplemental Figure S1***

Supplement: Supplementary file 1 [file cells-09-01142-s001.zip › Figure S1.pdf]

|            |   |   |   |   |
|------------|---|---|---|---|
| Mock       | + | + | + | - |
| Myc-BECN1  | - | + | - | + |
| Flag-TRAF6 | - | - | + | + |

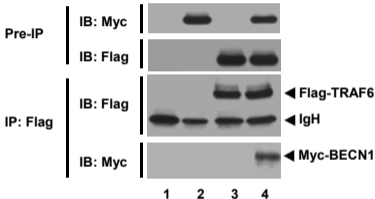

***Supplemental Figure S2***

Supplement: Supplementary file 1 [file cells-09-01142-s001.zip › Figure S2.pdf]

**A**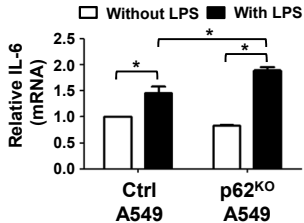**B**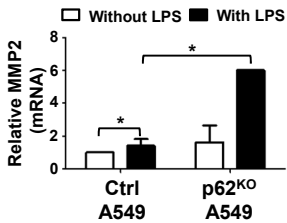**C**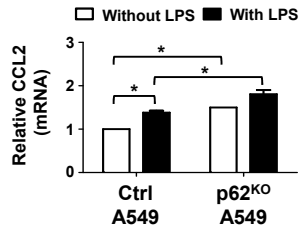

***Supplemental Figure S3***

Supplement: Supplementary file 1 [file cells-09-01142-s001.zip › Figure S3.pdf]

**A**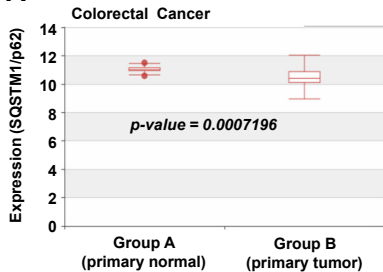**C**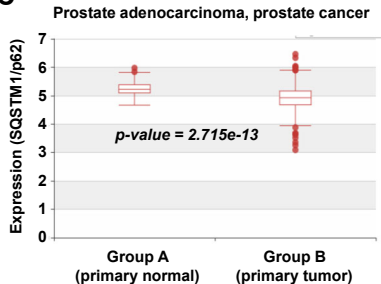**B**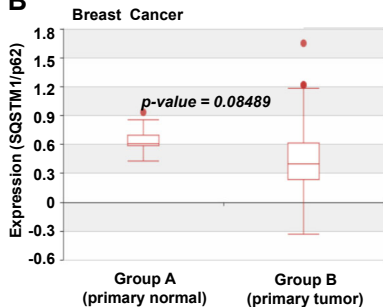**D**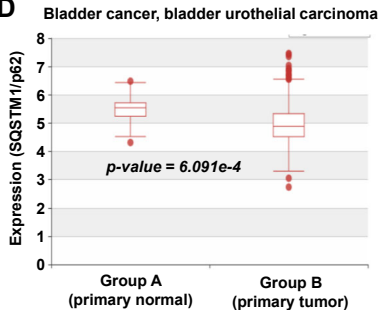

Supplement: Supplementary file 1 [file cells-09-01142-s001.zip › Figure S4.pdf]

**A**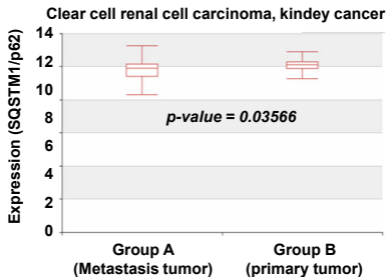**B**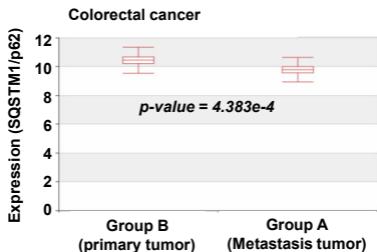

**Supplemental Figure S5**

Supplement: Supplementary file 1 [file cells-09-01142-s001.zip › Figure S5.pdf]
